# Identification of expanded and interrupted *ATXN2* repeat expansions in Parkinson’s disease and Lewy Body Dementia cohorts

**DOI:** 10.1038/s41531-025-01188-5

**Published:** 2025-11-27

**Authors:** Longfei Wang, Michael Milton, Liam G. Fearnley, Oneil G. Bhalala, Melanie Bahlo, Haloom Rafehi

**Affiliations:** 1 Genetics and Gene Regulation Division, The Walter and Eliza Hall Institute of Medical Research, Parkville, VIC Australia; 2 Department of Medical Biology, The University of Melbourne, Parkville, VIC Australia; 3 Bioinformatics Division, The Walter and Eliza Hall Institute of Medical Research, Parkville, VIC Australia; 4 Department of Neurology, Melbourne Brain Centre at The Royal Melbourne Hospital, University of Melbourne, Parkville, VIC Australia

**Keywords:** Genomics, Parkinson's disease

## Abstract

Repeat expansions (REs) may be Parkinson’s disease (PD) risk factors. We screened whole genome sequencing data from the AMP PD Lewy Body Dementia (LBD) and PD cohorts for 37 REs associated with neurological disorders, and identified both interrupted and uninterrupted REs in *ATXN2* in 4/2431 PD and 2/2468 LBD cases, but none in controls. These findings support pleiotropy for certain REs in PD.

Parkinson’s disease (PD) is a debilitating neurodegenerative disorder with a strong genetic component. Common genetic risk factors for sporadic PD have been identified at 78 independent genome-wide significant loci in a recent multi-ethnic genome-wide association study (GWAS)^1^.

Repeat expansions (REs) of short tandem repeats (STRs) are important genetic causes of neurodegenerative disorders, collectively referred to as repeat expansion disorders (REDs)^2,3^. While STR lengths are inherently variable between individuals, some STRs are pathogenic if expanded above a locus-specific threshold. Longer REs are often associated with increased disease severity and reduced age at onset. This phenotypic variation can result in substantial underdiagnosis. One recent population study screened whole genome sequencing (WGS) data and identified an allele frequency for known RED alleles of 1 in 283 individuals, which is higher than the approximate 1 in 3000 individuals who have a RED diagnosis, suggesting underdiagnosis, incomplete penetrance, or both^4^.

While no REs are known to uniquely cause PD, a RE is TAF1, is associated with an X-linked dystonia-parkinsonism (XDP) disorder in individuals from the Philippines^5^. Parkinsonism is observed as a symptom of some REDs, such as Spinocerebellar ataxia 2 (SCA2), which is caused by REs greater than the pathogenic threshold of 33 pure CAG repeats in *ATXN2*, without any interruptions with alternative base pairs in the CAG motif sequence (schematic of interrupted alleles illustrated in Fig. 1A)^3^. Disease pleiotropy has been previously described for REs in *ATXN2*, with intermediate expansions between 30-32 CAG repeats enriched in amyotrophic lateral sclerosis (ALS) cohorts^6,7^. Notably, REs greater than 32 repeats in *ATXN2*, in which the DNA sequence is interrupted with CAA repeats, have been observed in multiple PD studies, although reports are conflicting^8–10^. Other reports also implicate REs in *RFC1* in PD^11,12^. One limitation of previous studies is the lack of population prevalence data for REs.

**Fig. 1:**
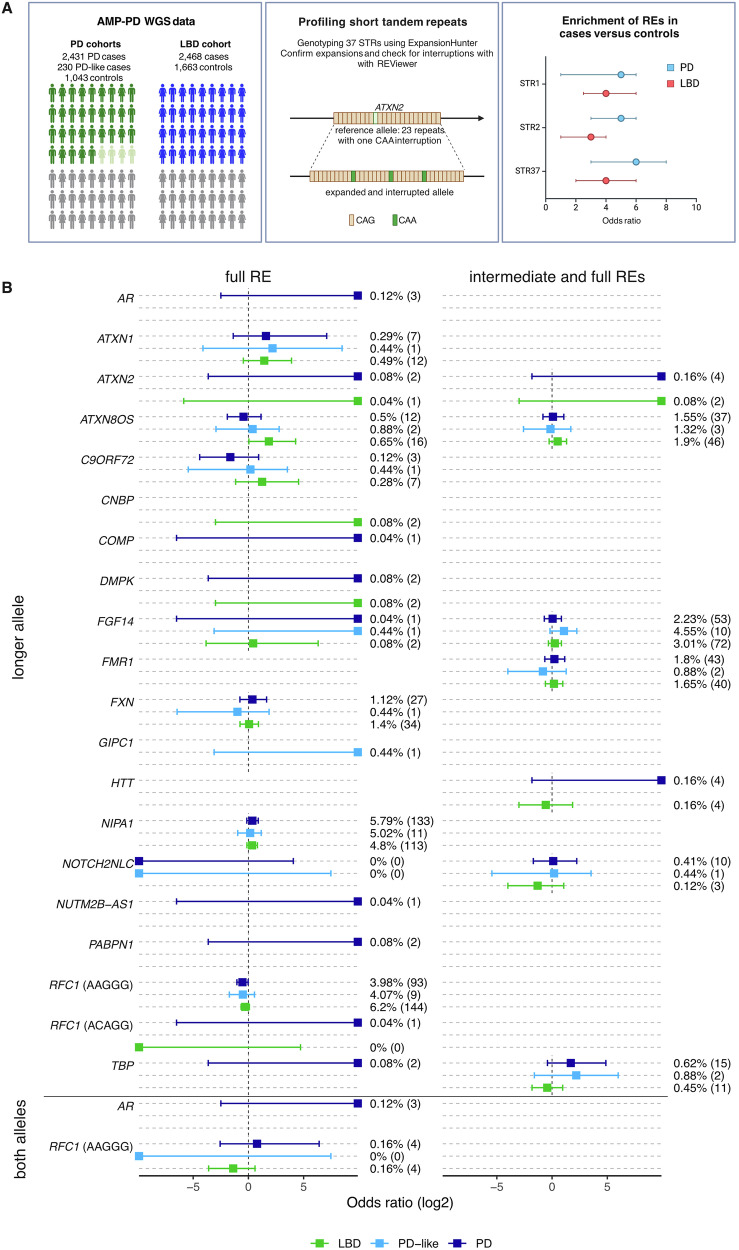
Study design and RE odds ratios (ORs). **A** Study design and overview. **B** Bars indicate 95% confidence intervals. ORs to the far right are infinite ORs as there are 0 samples in the controls, while those on the far left are infinite ORs as the cases are 0. ORs for both alleles were only calculated for recessive REDs. Calculations for intermediate thresholds also include REs that are greater than the pathogenic threshold. The percentage carrier frequency and number of carriers (indicated in brackets) are listed on the right-hand side of the confidence intervals.

Genotyping STRs in short-read sequencing data is now both feasible and reliable for most of the RED loci, due to advances in bioinformatics methods, which have been validated against current gold standard lab-based methods^3,4^.

Our study into the contributions of RE in PD was performed on a large European cohort consisting of WGS from 7835 individuals from the Accelerating Medicines Partnership programme for Parkinson’s Disease (AMP PD). Individuals of non-European ancestry were excluded from analysis due to small sample sizes, which is a study limitation. The combined PD cohorts consisted of 2431 PD cases, 230 PD-like cases (including disorders such as multiple system atrophy and progressive supranuclear palsy) and 1043 controls (Fig. 1A). An additional Lewy Body Dementia (LBD) cohort from the AMP PD comprised 2468 cases and 1663 controls. We compared estimated frequencies of genotypes determined by the Genome Aggregation Database (gnomAD) for 7487 European individuals, although not all loci are catalogued.

Genotyping was performed on a short list of 37 STRs using ExpansionHunter^13^. Alleles are referred to as REs if the genotype is larger than the reported number of repeats required to cause disease, i.e., the pathogenic threshold (Supplementary Table 1). A secondary, intermediate threshold was also applied to 10 loci based on previous reports that a shorter RE can also cause disease, typically with incomplete penetrance (Supplementary Table 1). Across both PD and LBD cohorts, we identified a combined 996 REs in the longer allele in the combined cases and controls in 19 genes (Supplementary Fig. 1), with REs in *ATXN1*, *ATXN2*, *HTT*, *DM1*, *NIPA1* and *RFC1* being the most frequent (Supplementary Table 2 and Supplementary Table 3). A further 464 REs in the intermediate range in six genes were also detected. We also observed 19 biallelic or hemizygous carriers of REs in *RFC1* (16) and *AR* (3 hemizygous males), both of which cause recessive REDs (Supplementary Fig. 2). Using Fisher’s exact test, no statistically significant enrichment in cases versus controls was observed following false discovery rate correction (Fig. 1B), also reflecting the modest sample size for detection of rare REs.

The CAG RE in *ATXN2* is the most widely reported STR associated with PD^9,14^. We observe expansions with >32 repeats in 0.16% (4/2431) of PD cases, 0.08% (2/2468) of LBD cases, whereas no expansions observed in either the PD (0/1043) or LBD (0/1663) control cohorts (Fig. 2A). These RE genotype calls are high confidence genotypes confirmed with the REViewer tool whereby the presence of reads with the repeat motif are visually confirmed to span the full STR (Supplementary Fig. 3). In gnomAD, 0.03% (3/9487) of European individuals have intermediate *ATXN2* expansions between 33–34 repeats. *ATXN2* REs >32 were found in 32/59,564 (0.05%) individuals of European ancestry in the 100,000 Genomes Project and TOPMed cohorts^4^. When considering only full REs > 34 repeats, the incidence is 0.08% (2/2,431) of PD cases, 0.04% (1/2,468) of LBD cases, and 0% in the PD and LBD controls, 0% in gnomAD and 10 in 59,564 (0.02%) in the combined 100,000 Genomes Project and TOPMed cohort^4^. Inspection of the *ATXN2* locus with REViewer identified that five of the six expanded alleles identified in the PD and LBD cohorts were interrupted with a CAG > CAA change (Supplementary Fig. 3). One PD case had 34 uninterrupted repeats, which is in the range for incompletely penetrant SCA2 (Table 1). Two of the PD cases are carriers of incompletely penetrant PD risk alleles. The small number of cases precluded identification of associations between *ATXN2* REs and PD/LBD clinical characteristics (Table 1). In gnomAD, we identified two interrupted REs with 33 repeats and one uninterrupted RE with 34 repeats. While these findings do not reach statistical significance due to the small study sample size, they are consistent with previous reports that expanded and interrupted *ATXN2* REs are genetic contributors to PD risk. This is the first study to report expanded, interrupted *ATXN2* alleles in LBD. Characterisation of the longer allele across all individuals in the PD and LBD cohorts showed that the most common allele length was 22 repeats (Fig. 2B). Next, we took a random stratified sample from the length distribution of the longer allele, examining 106 alleles to determine whether interruptions in the *ATXN2* STR are common. We found that all but two alleles had at least one CAG > CAA interruption (Fig. 2C).

**Fig. 2:**
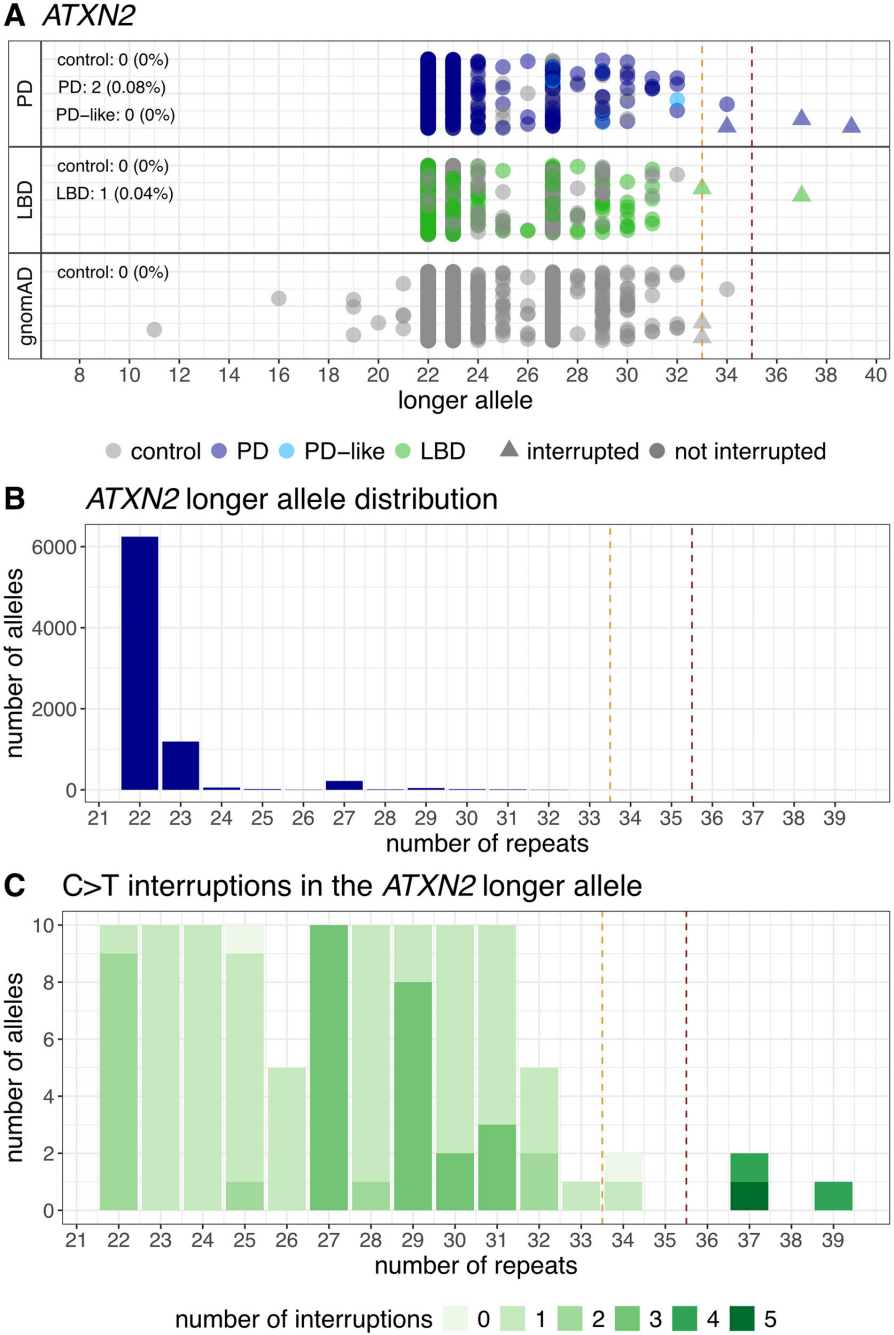
CAG REs detected in *ATXN2.* **A** Swimlane plot of the number of repeats in the longer allele in the PD, LBD and gnomAD cohorts. Triangles indicates REs with confirmed interruptions (only REs >= the intermediate threshold of 33 repeats were screened). Percentages indicate the number of individuals with a RE >= the pathogenic threshold of 35 repeats. **B** Distribution of the longer allele repeat size in all samples from PD and LBD cohorts. **C** Profiling CAG > CAA interruptions in a randomly generated and stratified subset of 106 individuals from the PD and LBD cohorts. Red dashed line: the pathogenic threshold at 35 repeats. Orange dashed line: pathogenic threshold at 33 repeats.

**Table 1:** Summary of REs in *ATXN2*

ID	Participant 1	Participant 2	Participant 3	Participant 4	Participant 5	Participant 6
Number of repeats	39	37	37	34	34	33
Number of interruptions (CAG > CAA)	4	5	4	0	1	1
Cohort	HBS	LBD	PDBP	PDBP	PDBP	LBD
Diagnosis	PD	LBD^	PD	PD	PD	LBD
Age range (y)	50-59	60-69	60-69	70-79	60-69	70-79
Family history of PD	Unknown	No	No	Yes	Yes	No
Carrier status*	No	No	GBA (N370S, rs76763715), APOE (E3/E4)	LRRK2 (G2019S, rs34637584)	No	No
MDS-UPDRS I	1	NA	3	6	19	NA
MDS-UPDRS II	6	NA	8	3	25	NA
MDS-UPDRS III	18	NA	11	24	31	NA
MDS-UPDRS IV	2	NA	0	4	0	NA
Cognitive impairment	Nil (MMSE = 30)	NA	Mild (MoCA = 25)	Nil (MoCA = 29)	Mild (MoCA = 19)	NA
Olfactory impairment	NA	NA	Severe (UPSIT = 11)	Nil (UPSIT = 37)	Nil (UPSIT = 35)	NA

*carrier status as reported by AMP PD. NA - data not available, ^pathologically confirmed (moderate plaques (CERAD = C); extensive tangles (Braak NFT = 5), DNA obtained from brain tissue). HBS cohort scores are UPDRS, not MDS-UPDRS.

Recent studies have suggested that biallelic *RFC1*-AAGGG REs are a rare cause PD, finding three biallelic carriers in a Finnish cohort of 569 individuals with PD (0.53%)^15^, an additional three cases in a follow up study in a Finnish cohort of 273 early-onset PD cases (1.1%) and four biallelic PD cases in the Parkinson’s Progression Markers Initiative (PPMI) cohort looking at 903 (0.44%) non-Finnish Europeans with PD, with none in controls^16^. The PPMI is also a subset of the AMP PD and is included in the present study. Although biallelic individuals are observed in this study, with 0.16% (4/2431) identified in the PD cohort, 0/230 in the PD-like cohort and 0.16% (4/2468) in the LBD cohort, biallelic expansions were also present in control cohorts: 0.1% (1/1043) in PD controls and 0.42% (7/1663) in LBD controls, and 0.11% (8/7487) in gnomAD (example REViewer plots in Supplementary Fig. 4). Therefore, no enrichment was observed. This is consistent with reports of 44 biallelic REs in *RFC1* in 29,496 (0.15%) European individuals in the 100,000 Genomes Project^4^. We note the discrepancy between the aforementioned PPMI study which identified four biallelic carriers. In our study, we identified three of the four cases from PPMI - the fourth case was excluded due to non-European ancestry. Furthermore, although recent studies show high concordance between biallelic AAGGG repeats, as determined by ExpansionHunter and REViewer, and confirmed by PCR^17–19^, we acknowledge that we cannot confirm the purity of the AAGGG alleles with short-read sequencing data. Hence, some of the biallelic cases may be false positives.

We observe 24 individuals with CAG REs >38 repeats in *ATXN1* in cases and controls, with no significant enrichment (Fig. 1). While uninterrupted CAG REs >38 repeats typically indicate SCA1, all REs observed contain at least 2 C > A interruptions, which negates the RE pathogenicity (Supplementary Table 1). Similarly, many REs were observed in *HTT* and *ATXN8OS* in cases and controls, with no significant enrichment. Other REs, such as in the genes *GIPC1, AR, CNBP* and *DMPK* were observed in cases but not controls, with very low incidence and no conclusions can be drawn.

To determine whether REs are enriched in PD and LBD overall, we performed Fisher’s Exact test on REs on three groups of data: a merge of (1) all loci, (2) all ataxia loci and (3) all non-ataxia loci. No significant associations were identified. There was no enrichment of REs in young-onset (<50 years) PD or LBD compared to late-onset, although this analysis was limited due to small sample sizes in the early onset groups. Meta-analysis for RE enrichment across the PD and LBD cohorts did not identify any significant results.

In conclusion, we identify REs in both PD and LBD cohorts, however there is no overall statistically significant RE enrichment in cases compared to controls. Several recent studies have shown that the prevalence of disease-causing REs is likely much higher than previously anticipated in general populations, which likely accounts for some of the associations of REs with PD observed in previous studies. The co-occurrence of the *ATXN2* RE with known, incompletely penetrant PD SNPs in two cases suggests a polygenic role for the RE in PD. Our finding of expanded and interrupted *ATXN2* alleles in the PD cases but not controls follows the trend reported in the literature, although larger cohorts are required to replicate this finding. Furthermore, we report for the first time interrupted *ATXN2* REs in LBD cases, suggesting a potential shared genetic mechanism.

## Methods

### Cohorts

The Walter and Eliza Hall Institute of Medical Research Human Research Ethics Committee (HREC 22/19) approved the study and all the procedures undertaken were in accordance with the ethical standards of the responsible committees. All components of this study were conducted in accordance with the principles embodied within the Declaration of Helsinki.

This study was performed on the tier two data from the Accelerating Medicines Partnership programme for Parkinson’s Disease (AMP PD, https://amp-pd.org/). Whole genome sequencing was performed by Macrogen and the Uniformed Services University of Health Sciences using the Illumina HiSeq XTen sequencer and was aligned to the GRCh38 reference genome. All primary analysis was performed within the Terra platform. For this study, we accessed WGS and phenotypic data from the LBD cohort and five PD cohorts: HBS, PDBP, PPMI, STEADY-PD3 and SURE-PD. Analysis was restricted to individuals of European ancestry due to population size constraints. Ancestry analysis was performed using reference populations from the 1000 Genomes Project^20^ and a cohort of Ashkenazi Jewish individuals^21^. The cohorts were merged with PLINK after excluding variants with a MAF < 0.1 and LD pruning and excluding mismatching SNPs. In addition, 402 controls from the PD cohorts were reported to have a family history of PD and were excluded from further analysis. Only unrelated individuals were included for further analysis. Relatedness analysis was performed with KING (v2.3.0). First, the merged pgen format genotype data were converted to bed format and pruned to exclude alleles with MAF < 0.05 using plink2 (v2.00a3.7LM). Next, KING was run to identify individuals with up to fourth-degree relatives. Related individuals were removed using a minimum weighted vertex cover method (MWVC). Priority was given to the samples in relatedness clusters based on their disease status in the following order: PD cases > PD-like cases > controls. As a result, 174 individuals were excluded from the PD cohorts (146 controls, 11 PD-like and 17 PD), and 71 from the LBD cohort (63 controls, 8 LBD cases).

In total, the study included 7835 individuals of European ancestry, of which the combined PD cohorts consisted of 2431(1524 male, 907 female) PD cases, 230 (136 male, 94 female) PD-like cases (including disorders such as multiple system atrophy and progressive supranuclear palsy) and 1,043 (469 male, 574 female) controls, and the LBD cohort with 2,468 (1555 males and 913 females) cases and 1663 (841 males and 822 females) controls.

STR genotype distribution data were obtained from gnomAD from 7487 non-Finnish European individuals as an additional control cohort. GnomAD contains genotype distributions for all loci included in this study, except for *FGF14*, *RILPL1*, *THAP11*, *ZFHX3* and *TAF1*. Data from gnomAD was generated using ExpansionHunter v5.0.0.

### Repeat expansion genotyping

Repeat expansion genotyping was performed within the Terra platform using ExpansionHunter v5.0.0^13^. A short-list of 37 STRs (summarised in Supplementary Table 1) was curated on the following criteria: (1) the REDs are typically adult-onset disorders, (2) the main phenotype is a movement disorder or dementia. The final list consists of 31 autosomal dominant disorders, four autosomal recessive disorders, one X-linked dominant disorder and one X-linked recessive disorder. All alleles have a pathogenic threshold above which they are considered to be pathogenic REs. A secondary intermediate RE threshold was also applied to 10 loci based on reports that a shorter RE can also cause disease, typically with incomplete penetrance (Supplementary Table 1). For the *FGF14* locus, a lower pathogenic threshold was applied, as it has previously been shown that the genotype obtained from REs estimated from WGS data are highly accurate when repeats are <150 bp in length but is underestimated in size for larger REs with the discrepancy increasing exponentially with increasing RE size^4,22^. REViewer was also used to confirm the motif for some loci, and to eliminate false positive RE calls^23^.

Post-processing was performed on the ExpansionHunter calls to remove genotypes where the motif does not match the motif specified in the STR catalogue. This was performed using the package EH5_kmerfilter (https://github.com/bahlolab/EH5_kmerfilter). Briefly, this method used a kmer-based filtering to identify and exclude genotypes in which the dominant motif at a locus does not match the locus defined in the catalogue.

REs for a subset of loci (*ATXN1*, *ATXN2*, *ATXN8OS*, *C9ORF72*, *CNBP*, *DMPK*, *FMR1*, *HTT*, *RFC1*, *TBP*), which are known to have a high false positive rate or where interruptions are important above the pathogenic or intermediate threshold, were visually checked for false positives or interruptions with REViewer plots (examples in Supplementary Fig. 3 and 4). For example, *FMR1* is known to have a high false positive rate with ExpansionHunter; therefore, all REs above the intermediate threshold were checked for false positives. All individuals carrying at least one copy of the AAGGG allele in *RFC1* were screened with REViewer, due to the high incidence of false negatives and positives for this STR.

Genotypes that are shorter than the read length (<150 bp) and have spanning reads are exact genotypes, as confirmed by the REViewer plots. However, STRs larger than 150 bp are estimates only, and not exact genotypes.

### Statistical analysis

Statistical analysis and graphical assessment were performed in R (v4.4.1). Fisher’s Exact test was used to determine potential enrichment of REs in cases versus controls for both the AMP PD and LBD cohorts. Enrichment was tested in the following ways in both the AMP PD and LBD cohorts:

1. In individual RE loci with dominant and recessive models and accounting for sex for X-linked loci,
  - All cases versus controls
  - All cases versus controls <50 years at baseline
  - All cases versus controls >=50 years old at baseline
  - In cases (PD, PD-like and LBD) only, comparing RE enrichment in early-onset (<50 years) versus late onset (>=50 years).
2. Group analysis in which loci are grouped into the following groups in cases versus controls:
  - all loci,
  - a grouped analysis of all ataxia RE loci and
  - a grouped analysis of all non-ataxia RE loci.

Differences in the genotyping methods used for the gnomAD STR calls and the AMP PD and LBD cohort calls precluded statistical analysis of STR frequencies from both cohorts.

Multiple test correction was performed using the False Discovery Rate method^24^. Meta-analysis was performed on the individual locus analysis with all samples for cases versus controls using Fisher’s method for combining p-values.

## Supplementary information

Supplementary Fig.

Supplementary table

## Data Availability

Access to the AMP PD data is available through the Terra platform (https://amp-pd.org/register-for-amp-pd). Individual-level genotype data, generated from the AMP PD, have been made accessible to the broader scientific community through the Terra workspace (https://app.terra.bio/#workspaces/bahlo_lab_amp_pd/MJFF-021399/files).

## Code availability

All scripts used for this study are available at: https://github.com/bahlolab/PD_repeat_expansions/.

## References

[1] Kim, J. J. et al. Multi-ancestry genome-wide association meta-analysis of Parkinson’s disease. *Nat. Genet.* **56**, 27–36 (2024). 38155330 10.1038/s41588-023-01584-8 PMC10786718

[2] Depienne, C. & Mandel, J.-L. 30 years of repeat expansion disorders: What have we learned and what are the remaining challenges? *Am. J. Hum. Genet.* **108**, 764–785 (2021). 33811808 10.1016/j.ajhg.2021.03.011 PMC8205997

[3] Rafehi, H., Bennett, M. F. & Bahlo, M. Detection and discovery of repeat expansions in ataxia enabled by next-generation sequencing: present and future. *Emerg. Top. Life Sci.*10.1042/ETLS20230018 (2023). 10.1042/ETLS20230018 PMC10754322 37733280

[4] Ibañez, K. et al. Increased frequency of repeat expansion mutations across different populations. *Nat. Med.* **30**, 3357–3368 (2024). 39354197 10.1038/s41591-024-03190-5 PMC11564083

[5] Bragg, D. C. et al. Disease onset in X-linked dystonia-parkinsonism correlates with expansion of a hexameric repeat within an SVA retrotransposon in TAF1. *Proc. Natl. Acad. Sci. USA.* **114**, E11020–E11028 (2017). 29229810 10.1073/pnas.1712526114 PMC5754783

[6] Elden, A. C. et al. Ataxin-2 intermediate-length polyglutamine expansions are associated with increased risk for ALS. *Nature* **466**, 1069–1075 (2010). 20740007 10.1038/nature09320 PMC2965417

[7] Henden, L. et al. Short tandem repeat expansions in sporadic amyotrophic lateral sclerosis and frontotemporal dementia. *Sci. Adv.* **9**, eade2044 (2023). 37146135 10.1126/sciadv.ade2044 PMC10162670

[8] Ross, O. A. et al. Ataxin-2 repeat-length variation and neurodegeneration. *Hum. Mol. Genet.* **20**, 3207–3212 (2011). 21610160 10.1093/hmg/ddr227 PMC3140823

[9] Casse, F. et al. Detection of ATXN2 expansions in an exome dataset: an underdiagnosed cause of Parkinsonism. *Mov. Disord. Clin. Pr.* **10**, 664–669 (2023). 10.1002/mdc3.13699 PMC10105108 37070044

[10] Wang, L. et al. Large-scale assessment of polyglutamine repeat expansions in Parkinson disease. *Neurology* **85**, 1283–1292 (2015). 26354989 10.1212/WNL.0000000000002016 PMC4617164

[11] Ylikotila, P. et al. Association of biallelic RFC1 expansion with early-onset Parkinson’s disease. *Eur. J. Neurol*. 10.1111/ene.15717 (2023). 10.1111/ene.15717 36705320

[12] Schüle, B. et al. Parkinson’s disease associated with pure ATXN10 repeat expansion. *NPJ Parkinsons Dis.* **3**, 27 (2017). 28890930 10.1038/s41531-017-0029-x PMC5585403

[13] Dolzhenko, E. et al. ExpansionHunter: a sequence-graph-based tool to analyze variation in short tandem repeat regions. *Bioinformatics* **35**, 4754–4756 (2019). 31134279 10.1093/bioinformatics/btz431 PMC6853681

[14] Kim, J.-M. et al. Importance of low-range CAG expansion and CAA interruption in SCA2 Parkinsonism. *Arch. Neurol.* **64**, 1510–1518 (2007). 17923635 10.1001/archneur.64.10.1510

[15] Kytövuori, L. et al. Biallelic expansion in RFC1 as a rare cause of Parkinson’s disease. *NPJ Parkinsons Dis.* **8**, 6 (2022). 35013364 10.1038/s41531-021-00275-7 PMC8748909

[16] Alvarez Jerez, P. et al. Profiling complex repeat expansions in RFC1 in Parkinson’s disease. *NPJ Parkinsons Dis.* **10**, 108 (2024). 38789445 10.1038/s41531-024-00723-0 PMC11126591

[17] Rafehi, H. et al. A prospective trial comparing programmable targeted long-read sequencing and short-read genome sequencing for genetic diagnosis of cerebellar ataxia. *Genome Res* **35**, 769–785 (2025). 40015980 10.1101/gr.279634.124 PMC12047251

[18] Davies, K. C. et al. Comprehensive characterisation of the RFC1 repeat in an Australian cohort. *Cerebellum* **24**, 111 (2025). 40481300 10.1007/s12311-025-01867-2

[19] Sullivan, R. et al. *RFC1*repeat expansion analysis from whole genome sequencing data simplifies screening and increases diagnostic rates. *bioRxiv* 2024.02.28.24303510 10.1101/2024.02.28.24303510 (2024).

[20] 1000 Genomes Project Consortium. et al. A global reference for human genetic variation. *Nature* **526**, 68–74 (2015). 10.1038/nature15393 PMC4750478 26432245

[21] Need, A. C., Kasperaviciute, D., Cirulli, E. T. & Goldstein, D. B. A genome-wide genetic signature of Jewish ancestry perfectly separates individuals with and without full Jewish ancestry in a large random sample of European Americans. *Genome Biol.* **10**, R7 (2009). 19161619 10.1186/gb-2009-10-1-r7 PMC2687795

[22] Rafehi, H. et al. An intronic GAA repeat expansion in FGF14 causes the autosomal-dominant adult-onset ataxia SCA50/ATX-FGF14. *Am. J. Hum. Genet.* **110**, 105–119 (2023). 36493768 10.1016/j.ajhg.2022.11.015 PMC9892775

[23] Dolzhenko, E. et al. REViewer: haplotype-resolved visualization of read alignments in and around tandem repeats. *Genome Med.* **14**, 84 (2022). 35948990 10.1186/s13073-022-01085-z PMC9367089

[24] Benjamini, Y. & Hochberg, Y. Controlling the false discovery rate: A practical and powerful approach to multiple testing. *J. R. Stat. Soc. Ser. B Stat. Methodol.* **57**, 289–300 (1995).

