# Supplementary material for: Identification of expanded and interrupted ATXN2 repeat expansions in Parkinson’s disease and Lewy Body Dementia cohorts
Source: NPJ Parkinsons Dis. 2025 Nov 27;11:341. doi: 10.1038/s41531-025-01188-5 (PMC12661016; doi:10.1038/s41531-025-01188-5)
Supplement: Supplementary file 1 — Supplementary Fig. [file 41531_2025_1188_MOESM1_ESM.docx]

**Supplementary materials**


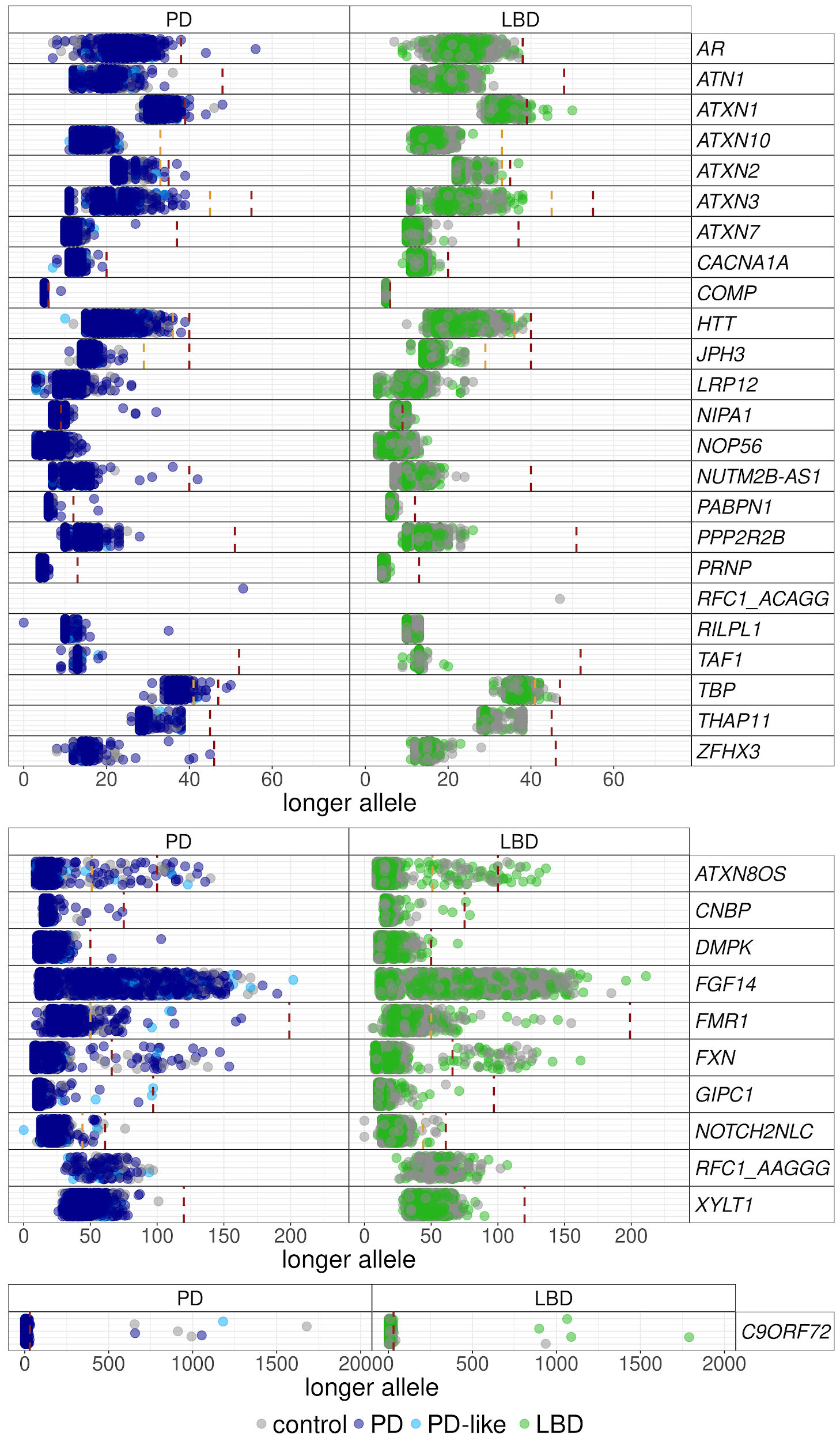


**Supplementary Figure 1. Swimlane plots showing the longer allele repeat numbers for the PD (blue), PD-like (light blue) and LBD (green) cases and controls (grey), as determined by ExpansionHunter.** Red dashed line: the pathogenic threshold. Orange dashed line: intermediate threshold.


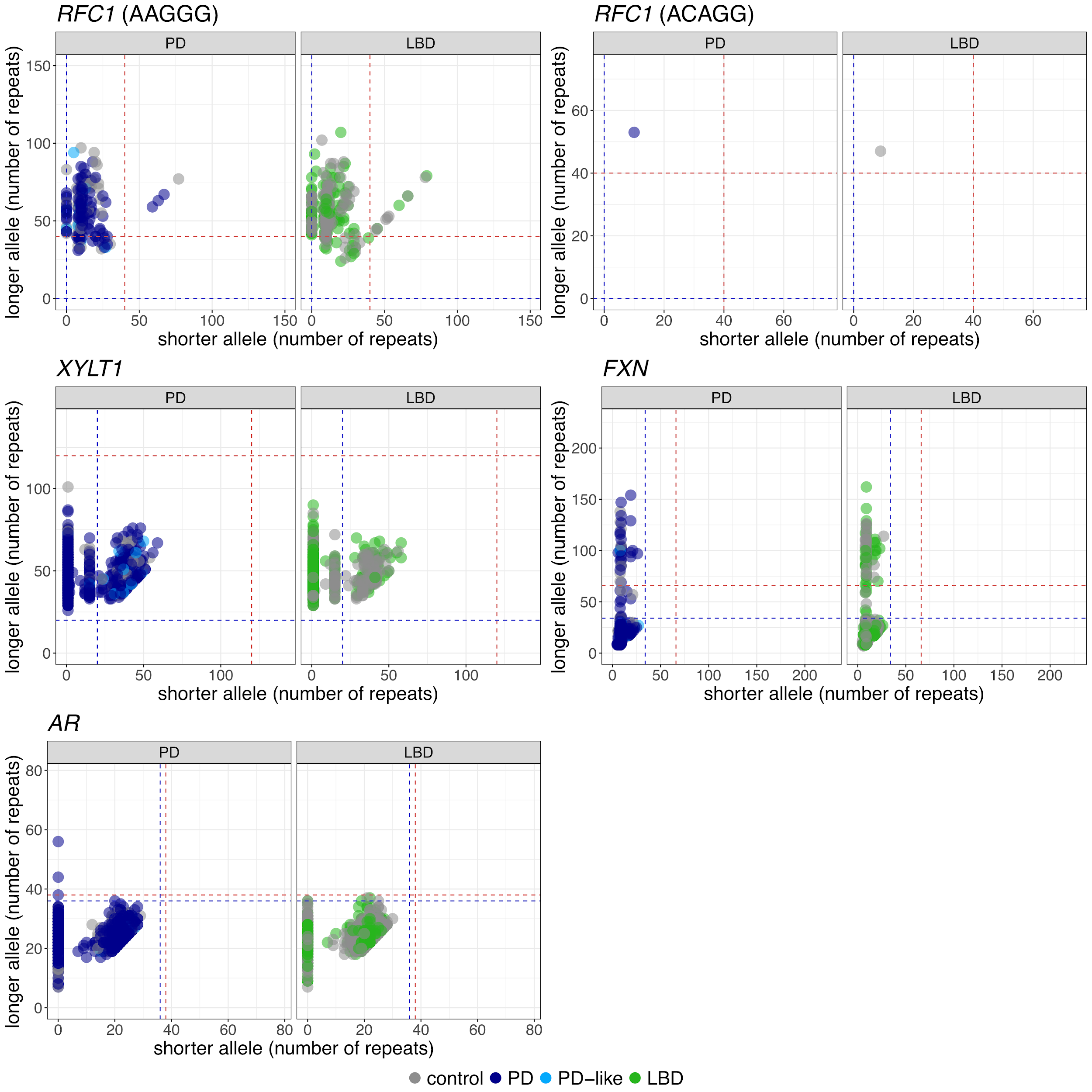


**Supplementary Figure 2.** Shorter and longer allele sizes estimated from ExpansionHunter for all recessively inherited disorders.

**Participant 1**

**Participant 2**

**Participant 3**

**Participant 4**

**Participant 5**

**Participant 6**

**Supplementary Figure 3. REViewer plots of the expanded *ATXN2* allele for all participants summarised in Table 1.** The red segments indicate the reads mapping to the STR (GTC corresponds to CAG on the coding strand). Interruptions within the STR are shown as the letter T (C>T in the reference genome, corresponds to G>A on the coding strand). The blue segments indicate reads mapping to the flanking DNA. The numbers above each figure indicate the allele size genotyped by ExpansionHunter.

***ATXN1***

***ATXN8OS***

***C9ORF72***

***CNBP***

***DMPK***

***FMR1***

******

***HTT***

******

***RFC1***

***TBP***

**Supplementary Figure 4. Example REViewer plots of the expanded alleles for *ATXN1*, *ATXN8OS*, *C9ORF72*, *CNBP*, *DMPK*, *FMR1*, *HTT*, *RFC1*, *TBP*.** The red or green segments indicate the reads mapping to the STR. Interruptions within the STR are shown as the letter representing the nucleotide (A,C,G,T) that is interrupting the motif. The blue segments indicate reads mapping to the flanking DNA. The numbers above each figure indicate the allele size genotyped by ExpansionHunter.
